# Keeping *Lagocephalus sceleratus* off the Table: Sources of Variation in the Quantity of TTX, TTX Analogues, and Risk of Tetrodotoxication

**DOI:** 10.3390/toxins13120896

**Published:** 2021-12-13

**Authors:** Georgios Christidis, Manolis Mandalakis, Thekla I. Anastasiou, George Tserpes, Panagiota Peristeraki, Stylianos Somarakis

**Affiliations:** 1 Institute of Marine Biological Resources and Inland Waters, Hellenic Centre for Marine Research (HCMR), 71500 Heraklion, Greece; gtserpes@hcmr.gr (G.T.); notap@hcmr.gr (P.P.); somarak@hcmr.gr (S.S.); 2 Biology Department, University of Crete, 70013 Heraklion, Greece; 3 Institute of Marine Biology, Biotechnology and Aquaculture, Hellenic Center of Marine Research (HCMR), 71500 Heraklion, Greece; theanast@hcmr.gr

**Keywords:** *Lagocephalus sceleratus*, Mediterranean Sea, tetrodotoxin, LC-MS/MS, linear models

## Abstract

The invasion of the tetrodotoxin (TTX)-bearing silver-cheeked toadfish and potential poisoning due to its consumption (tetrodotoxication) threatens public safety in the Mediterranean Sea. In this study, TTX and TTX analogues of *Lagocephalus sceleratus* (Gmelin, 1789) were measured using liquid chromatography tandem mass spectrometry (LC-MS/MS) in fish collected off the island of Crete (Southern Mediterranean). We tested the synergistic effect of a suite of factors potentially affecting toxins’ levels and tetrodotoxication risk using general and generalized linear models, respectively. The type of tissue, geographic origin (Cretan Sea, Libyan Sea), sex, and fish maturity stage were significant predictors of toxin concentrations. Mean TTX was higher in gonads and lower in muscles, higher in the Libyan Sea and in female fish, and lower in juvenile (virgin) fish. The concentration of TTX was also significantly and positively correlated with the concentration of several TTX analogues (4-epiTTX, 4,9-anhydroTTX, 11-deoxyTTX, 5,11/6,11-dideoxyTTX, 5,6,11-trideoxyTTX, 11-norTTX-6-ol). The analysis showed that fish originating from the Libyan Sea had significantly higher probability to cause tetrodotoxication in case of consumption. The variability explained by the models developed in this study was relatively low, indicating that toxin levels are hard to predict and the consumption of *L. sceleratus* should therefore be avoided.

## 1. Introduction

Tetrodotoxin (TTX) is a potent, non-protein, water-soluble, and heat-stable neurotoxin [1] that acts as a blocker in voltage-gated sodium channels, thus inhibiting nerve and muscle conduction [2]. It is present in a variety of aquatic and terrestrial organisms with pufferfishes (family: Tetraodontidae) being the most widely known TTX bearers [3]. The accumulation of TTX in animals plays numerous ecological roles [4], including its function as a defensive mechanism against predators [5], a predation mechanism [6], a pheromone [7], or an egg-protecting agent [8].

The origin of TTX in pufferfishes remains largely unclear, with many studies supporting the hypothesis that TTX is accumulated through the food chain (exogenous origin hypothesis) rather than being produced by endosymbiotic or parasitic bacteria inside the pufferfish body (endogenous origin hypothesis) [9]. Most data on TTX concentrations in pufferfish species come from the Indo-Pacific region [10,11,12,13] where these fishes are consumed and, historically, many TTX intoxications (tetrodotoxications) have occurred [14].

In nature, TTX has been found to coexist with 26 TTX analogues, many of which have been detected in pufferfishes [15]. These can be grouped into the following: (1) chemical equilibrium analogues (4-epiTTX and 4,9-anhydroTTX), (2) deoxy analogues (5-deoxyTTX, 11-deoxyTTX, 5,11-dideoxyTTX, 6,11-dideoxyTTX, and 5,6,11-trideoxyTTX), (3) 11-CH_2_OH-oxidized analogues (11-oxoTTX), and (4) C_11_-lacking analogues (11-norTTX-6(S)-ol and 11-norTTX-6(R)-ol) [16]. Studies on the biosynthesis and metabolism of TTX have suggested that deoxy analogues are precursors of TTX, and 4-epiTTX and 4,9-anhydroTTX are derivatives of epimerization and dehydration of TTX, whereas 11-oxoTTX and 11-norTTX-6-ol are oxidation metabolites of TTX [16,17]. In recent years, the detection and quantification of TTX and its analogues have been conducted using liquid chromatography tandem mass spectrometry (LC-MS/MS) [18], which has progressively replaced the mouse bioassay method (MBA) [19], as a more accurate and ethical alternative for toxicity assessments [20].

The present study aimed at assessing the levels of TTX and TTX analogues in the silver-cheeked toadfish *Lagocephalus sceleratus*, one of the worst invasive species in the Eastern Mediterranean [21]. *L. sceleratus* is a tetraodontid pufferfish of Indo-Pacific origin that entered into the Mediterranean Sea from the Red Sea through the Suez Channel (‘Lessepsian migrant’) [22]. It soon established abundant populations, particularly in the Eastern Mediterranean, with negative impacts on native biodiversity and local fisheries, while potential TTX poisoning due to its consumption threatens public safety [21]. Although legislation in the European Union [23,24] and Eastern Mediterranean countries (i.e., Egyptian legislation [25]; Turkish legislation [26]) prohibit the landing, trade, and consumption of *L. sceleratus* and its products, many poisonings and fatalities have already been reported from Eastern Mediterranean countries [27,28,29,30,31,32,33].

The urgent need of managing *L. sceleratus* populations, raising public awareness, and protecting human health has led to numerous studies along the Mediterranean coasts, which have investigated, compared, and proposed methodologies for monitoring the toxicity of this species [34,35,36,37,38,39,40,41,42]. In the latter investigations, the concentration of TTX in different tissues was quantified and often compared between sexes, fish sizes, and seasons. Most of these studies were based on a limited number of samples (e.g., three samples were analyzed in the study of Bane et al. [36]), whereas TTX analogues have rarely been measured [35,36,39,40]. Moreover, factors presumed to affect the toxicity of *L. sceleratus* (e.g., tissue, sex, season, fish size) have always been considered separately.

The aim of the present investigation was to assess the variability and to model the concentration of TTX and TTX analogues in *L. sceleratus*, as well as to estimate the risk of tetrodotoxication from consumption of its flesh, using an integrated approach that takes into account the synergetic effect of a suite of parameters, known to potentially affect the quantity of TTX in pufferfishes (tissue, sex, season, fish maturity stage, geographical origin) [43,44]). For this purpose, we used linear models applied to one of the largest sets of LC-MS/MS measurements ever attained for this species.

## 2. Results

### 2.1. TTX and TTX Analogues Levels in L. sceleratus Tissues

Tetrodotoxin and three of its analogues, namely 4-epiTTX, 11-norTTX-6-ol, and 11-deoxyTTX, were detected in all samples analyzed (*n* = 332), while detection of 5,11/6,11-dideoxyTTX, 4,9-anhydroTTX, and 5,6,11-trideoxyTTX was possible in 96%, 90%, and 79% of them, respectively (Table 1). Overall, the highest toxin levels in terms of mean concentration of total TTX (TTX + TTX analogues) were detected in the gonads (77.80 ± 98.73 μg g^−1^), followed by the liver (29.56 ± 59.92 μg g^−1^), skin (6.59 ± 7.35 μg g^−1^), and muscle (5.89 ± 8.49 μg g^−1^), while similar patterns were observed for the mean concentrations of individual toxins (Table 1). In all tissue types, TTX exhibited the highest concentration levels (3.76 ± 4.59 μg g^−1^–39.85 ± 45.45 μg g^−1^) followed by 11-deoxyTTX (1.38 ± 3.40 μg g^−1^–19.23 ± 40.62 μg g^−1^), whereas 5,6,11-trideoxyTTX had the lowest concentration (0.005 ± 0.04 μg g^−1^–1.46 ± 5.85 μg g^−1^).

**Figure 1:**
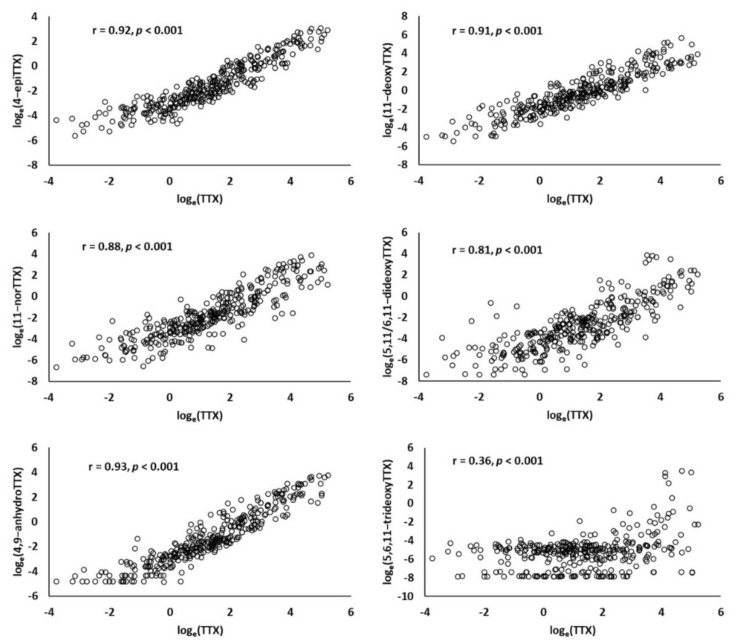
Scatterplots and Pearson correlation coefficients (r) between the concentration of TTX and concentrations of TTX analogues in *L. sceleratus* tissue samples.

The concentrations of TTX analogues were highly and significantly correlated with TΤΧ (Pearson correlation coefficients: r = 0.81–0.93, all *p* < 0.001) with the exception of 5,6,11-trideoxyTTX, which exhibited a weak correlation with TTX (r = 0.36, *p* < 0.001) (Figure 1). The 5,6,11-trideoxyTTX was the toxin with the lowest mean concentration (Table 1) and its levels were below the detection limit in 21% of the analyzed samples.

### 2.2. General Linear Models (GLMs) for TTX and TTX Analogues

The GLM analysis for TTX concentration revealed that the effects of TISSUE, AREA, SEX, and MATURITY were highly significant, whereas SEASON and SIZE were not significant at the 0.05 level (Table 2). The fitted model explained 46% of total variance, with TISSUE, AREA, SEX, and MATURITY accounting for 24%, 14%, 4%, and 3% of the total variance, respectively (Table 2).

**Figure 2:**
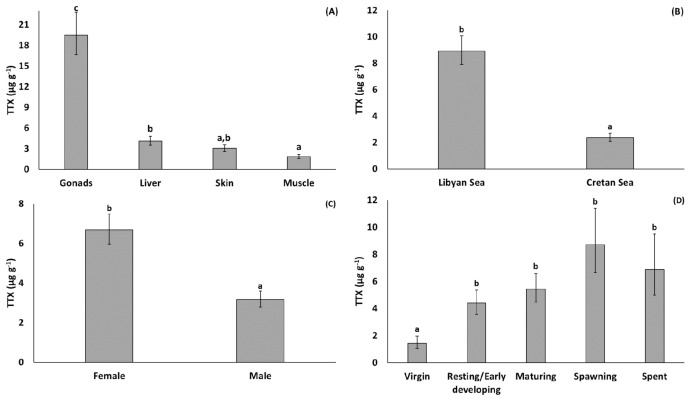
Least-square means (back-transformed) of TTX concentration for the effects of (**A**) TISSUE, (**B**) AREA, (**C**) SEX, and (**D**) MATURITY. Error bars represent standard errors (back-transformed). a < b < c: homogeneous groups (Bonferroni tests).

With regard to the effect of tissue type (Figure 2), the least-square mean concentration of TTX was significantly higher for gonads compared to the other tissues (back-transformed estimate: 19.46 µg g^−1^), and significantly higher for liver (4.08 µg g^−1^) compared to muscle (1.85 µg g^−1^). The least-square mean TTX for skin (3.02 µg g^−1^) did not differ significantly between muscle and liver (Figure 2). With regard to geographical origin, the least-square mean TTX was significantly higher for specimens collected from the Libyan Sea (8.91 µg g^−1^) than from Cretan Sea (2.37 µg g^−1^) (Figure 2). TTX was also significantly higher in females (6.68 µg g^−1^) than in males (3.16 µg g^−1^) (Figure 2). Finally, the TTX concentration was affected by fish reproductive state: the least-square mean TTX was significantly lower in virgin fish (stage 0: 1.44 µg g^−1^) than adults (stages 1–4: 4.39–8.70 µg g^−1^) and appeared to exhibit an increasing, albeit non-significant, trend across subsequent stages of gonadal maturity (from resting/early developing (stage 1) to spawning and post-spawning fish (stages 3 and 4) fish) (Figure 2).

In the analyses of relative concentrations of TTX analogues (Table 3), we used the TISSUE, AREA, SEX, and MATURITY as independent variables, i.e., the factors significantly affecting the absolute TTX concentration (see above). The analogue 5,6,11-trideoxyTTX, which presented the lowest concentrations (Table 1) and a poor correlation with TTX (Figure 1) was not considered further. All TTX analogue models were highly significant (*p* < 0.001) and adjusted-r^2^ values ranged from 17% to 34% (Table 3).

**Figure 3:**
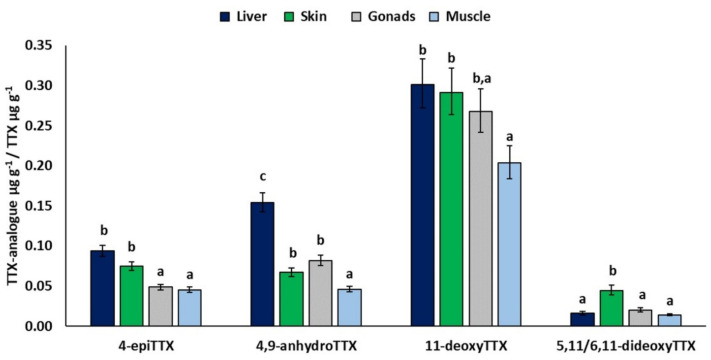
Significant effects of TISSUE. Least-square means (back-transformed) for relative concentration of TTX analogue from GLMs for 4-epiTTX, 4,9-anhydroTTX, 11-deoxyTTX, and 5,11/6,11-dideoxyTTX. Error bars represent standard errors (back-transformed). a < b < c: homogeneous groups (Bonferroni tests).

SEX had a significant effect in all models (Table 3). All least-square mean TTX analogue/TTX ratios were significantly higher in females than in males (not shown). Moreover, all ratios were found to be significantly affected by TISSUE except for 11-norTTX-6-ol. More specifically, the least-square mean of 4-epiTTX/TTX was significantly higher in liver (0.09) and skin (0.07) compared to gonads (0.05) and muscle (0.05), whereas the ratios for 4,9-anhydroTTX and 5,11/6,11-dideoxyTTX were significantly higher for liver (0.15) and skin (0.04), respectively, compared to other tissues (Figure 3). Furthermore, the mean 4,9-anhydroTTX/TTX was significantly higher in gonads (0.08) and skin (0.07) compared to muscle (0.05). Finally, the estimated mean relative concentration of 11-deoxyTTX was significantly lower in muscle (0.20) compared to liver (0.30) and skin (0.29) (Figure 3).

**Figure 4:**
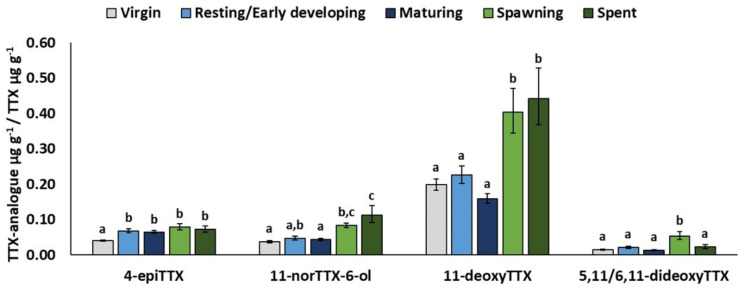
Significant effects of MATURITY. Least-square means (back-transformed) for relative concentration of TTX analogue from GLMs for 4-epiTTX, 11-norTTX-6-ol, 11-deoxyTTX, and 5,11/6,11-dideoxyTTX. Error bars represent standard errors (back-transformed). a < b < c: homogeneous groups (Bonferroni tests).

MATURITY significantly affected the relative concentrations of all TTX analogues except 4,9-anhydroTTX (Table 3). Specifically, the least-square mean 4-epiTTX/TTX was significantly lower in juveniles (stage 0: 0.04) than in adult fish (stages 1–4: 0.06–0.08) (Figure 4). On the other hand, the estimated relative concentrations of 11-norTTX-6-ol, 11-deoxyTTX, and 5,11/6,11-dideoxyTTX were, in general, significantly higher in spawning and post-spawning fish (maturity stages 3 and 4). Finally, AREA was only significant for 11-norTTX-6-ol and 4,9-anhydroTTX, which presented, similarly to TTX, higher (*p* < 0.05) relative concentrations in the Libyan Sea than in the Cretan Sea.

### 2.3. Tetrodotoxication Risk

Overall, 48% of the muscle samples analyzed were characterized as non-toxic and safe for consumption (i.e., TTX eq ≤ 2.2 µg g^−1^), whereas 52% were assigned as toxic. Furthermore, 95% and 5% of the toxic samples were slightly (2.2 µg g^−1^ < TTX eq ≥ 22 µg g^−1^) and moderately toxic (22 µg g^−1^ < TTX eq ≥ 220 µg g^−1^), respectively, while none was assigned as extremely toxic (TTX eq > 220 µg g^−1^). The fit of the binomial model to the assigned (0/1) values of the muscle samples (non-toxic/toxic) revealed that only the effect of AREA was statistically significant (Table 4). In fact, the probability of food poisoning after the consumption of a pufferfish meal was significantly higher, nearly double, for fish originating from the Libyan Sea compared to the Cretan Sea, although the confidence intervals of the Libyan Sea probability were wider (food poisoning probability: Libyan Sea = 0.87 (lower confidence limit = 0.66, upper confidence limit = 0.96]; Cretan Sea = 0.42 [lower confidence limit = 0.18, upper confidence limit = 0.71)).

## 3. Discussion

### 3.1. TTX and TTX Analogues Levels in L. sceleratus Tissues: Comparisons with Other Studies

To date, many studies conducted in the Mediterranean Sea have detected/quantified TTX levels in *L. sceleratus* tissues by using various analytical instruments and methods (summarized in Table 5). Among the systems used, those based on liquid chromatography and mass spectrometry are the most common, as LC-MS is recognized as the state of the art in the analysis of TTX and its analogues. Although previous studies provide valuable information regarding the toxicity of *L. sceleratus*, comparisons of their findings with our results (Table 5) are often not straightforward, mainly because of the wide differences in both the methodological approaches and sample sizes used (i.e., less than half of the number of specimens analyzed in the present study were analyzed in the majority of previous studies).

In our study, TTX was the major toxin of *L. sceleratus*, followed by 11-deoxyTTX, whereas 5,6,11-trideoxyTTX had the lowest concentration in all tissues. Similarly, Bane et al. [36] found that TTX and 11-deoxyTTX were among the most abundant analogues detected in all tissues, whereas 5,6,11-trideoxyTTX, although present with high concentrations in some samples, was completely absent in others. In contrast, Rodríguez et al. [35], Acar et al. [39], and Rambla-Alegre et al. [40] found that 5,6,11-trideoxyTTX was the major analogue detected in *L. sceleratus* tissues. These discrepancies can be attributed to the low number of specimens analyzed in previous studies, the different methods used for chemical analysis, and, particularly, the unavailability of a reliable 5,6,11-trideoxyTTX standard of high purity that would enable the unequivocal calibration of the LC-MS/MS detection systems. Overall, the dominance of TTX in the toxins’ composition has also been reported for many other pufferfish species (i.e., *Takifugu oblongus*, *Fugu nipholbes*, *Tetraodon nigoviridis*, *T. biocellatus*, and *F. poecilonotus*) [49,50,51].

The present study showed that, on average, gonads had the highest mean TTX concentration followed by the liver and then skin, whereas the lowest TTX concentration was detected in muscles. In terms of maximum TTX concentration, gonads ranked first, followed by liver, muscle, and skin. A similar pattern has also been observed in previous studies on *L. sceleratus* (those with more than one specimen analyzed, Table 5 [35,37,38,39]). Moreover, the intratissue pattern of TTX concentration in specimens from the Cretan and Libyan Seas is in line with that of other marine pufferfishes [3,12,52].

Although gonads seem to generally exhibit the highest TTX levels, Akbora et al. [42] found that the concentration of TTX was maximized in liver. This individual case may also be attributed to the small fish sample and the different methodology used. Interestingly, the maximum TTX concentrations that we recorded in gonads, liver, muscle, and skin were the highest among the other Mediterranean areas (Table 5). Overall, the ranges of TTX concentrations that we measured in all tissues are the widest ever reported for *L. sceleratus* in the Mediterranean, regardless of area and the method used. The wide ranges of TTX concentrations recorded in this study can be attributed, at least in part, to the larger number of specimens analyzed, which helped to reveal the high magnitude of variability in TTX concentration between individuals of this species.

### 3.2. Sources of Variation in TTX Levels

The present investigation attempted to provide a more comprehensive picture regarding the factors affecting toxicity in *L. sceleratus* by using a large sample size and applying an integrated, multiparametric approach (general linear models) to examine how the levels of TTX and its analogues are modulated. From the variables tested, TISSUE, AREA, SEX, and MATURITY stage significantly affected the TTX concentration. However, the general linear model explained a moderate (46%) amount of data variation. The type of tissue accounted for the largest part of explained variance, followed by geographical origin, sex, and maturity stage. These findings are largely in line with the study of Endo [43], who examined the TTX levels in Japanese pufferfishes (15 species) and concluded that TTX concentration is highly variable, both among individuals and among species, with mean TTX levels showing obvious differences between males and females as well as between different tissues, seasons, and sampling areas.

The results of the GLM (which take into account the effect of all predictors entering into the model) indicated that the mean TTX concentration was approximately five to ten times higher in gonads compared to other tissues, and about two times higher in the liver compared to muscle. The intratissue pattern of mean TTX predicted by the model was similar to that inferred from the mean TTX concentrations calculated from the raw data (Table 1) and discussed above. The GLM also showed that, on average, TTX concentration was approximately two times higher in females compared to males. This sex-related difference in TTX concentration is a common feature in pufferfishes [53,54]. It may be explained, at least in part, by the role of TTX as a male-attracting pheromone [7]. Furthermore, TTX is known to be transferred to pufferfish eggs, most likely acting as a defense mechanism of maternal origin, to protect the fertilized eggs and larvae from predation risk [55].

Concerning the effect of maturity stage, virgin fish (maturity stage 0) had almost four times lower estimated mean TTX concentration compared to actively spawning adult fish (maturity stage 3). Additionally, fish that had reached first maturity (stages 1–4) exhibited an increasing trend in mean TTX with stage of gonadal development (from the resting to the spawning stage). Similarly, Sabrah et al. [56], Katikou et al. [34], and Acar et al. [39] reported that small, immature *L. sceleratus* have lower TTX levels compared to mature individuals, with the former study also showing that TTX concentration increases with maturation stage of the gonad. Further support to these findings is provided by Noguchi and Arakawa [3] and Arakawa et al. [57] who suggest that, in the Indo-Pacific region, pufferfishes’ toxicity relates to gonadal activity, with female fish being more toxic during the spawning period, probably due to changes in the transfer and/or accumulation of TTX associated with ovarian maturation [44]. On the other hand, Kosker et al. [37] report that gonads of both adult male and female pufferfish are more toxic during the non-spawning period (autumn and winter) compared to the spawning season (spring and summer). Despite the fact that fish size (total length) did not enter significantly in the GLM for TTX, it should be noted here that SIZE is somehow related to MATURITY, because the maturity stage 0 (virgin fish, with significantly lower TTX concentration) encompassed only the smallest specimens in the fish sample (fish <383 mm total length). The same is true for SEASON. It is largely related to MATURITY, since maturity stages 3 (spawning) and 4 (spent) occur only in spring and summer, respectively, i.e., inside the limits of the species’ spawning period [58,59]. It was therefore not surprising that the most toxic fish in this study were caught in spring and summer, an observation that agrees with the findings of El-Sayed et al. [60] in the Red Sea and Akbora et al. [42] in Cyprus.

Following TISSUE, the factor AREA ranked second in explaining TTX variation, with fish originating from the Libyan Sea (south) having approximately four times higher levels of TTX compared to fish from the Cretan Sea (north). This regional disparity may reflect differences in diet composition. This is fairly likely given that the predominant theory explaining TTX (bio)accumulation in pufferfishes is through the food chain, in which the primary TTX-producing organisms are marine bacteria [3,9,61,62,63,64,65]. A preliminary analysis of gastrointestinal track contents of the specimens examined in the present study (Supplementary Materials Table S2) showed that prey composition differed significantly between the Libyan and Cretan Seas. In particular, gastropods, bivalves, and unidentified preys presented higher frequencies of occurrence in the gastrointestinal tracks of specimens collected from the Libyan Sea compared to the Cretan Sea. Although it is unknown whether the prey species found in this study contain TTX, this was an intriguing finding given that many gastropods and bivalve species are known TTX bearers [3,66]. The observed differences in gastrointestinal content composition provide some evidence that the diet of *L. sceleratus* may be responsible, at least in part, for the disparity in TTX concentration between the two areas.

Finally, according to Noguchi et al. [9], an additional source of TTX in pufferfish could be through the symbiosis with TTX-producing bacteria belonging to the intestinal microflora. The investigation of this important aspect was not possible in the present study. However, we expect that future metagenomic analysis of gastrointestinal microbiome of *L. sceleratus* will help to characterize the microbial community composition and elucidate whether the different toxicity levels between fish from the Cretan and Libyan Seas could be attributed to the symbiosis with different bacteria in the two areas.

Furthermore, as mentioned in Section 5.1, the Cretan and Libyan Seas present differences in their physical environment, which could also explain the regional differences in TTX levels. For example, temperature is higher in the Libyan Sea [67,68], which could enhance the production of TTX by endosymbiotic bacteria. In laboratory experiments Auawithoothij and Noomhorm [69] showed that temperature and salinity significantly affected the production of TTX in the bacterium *Shewanella putrefaciens* isolated from the pufferfish *Lagocephalus lunaris*.

Considerations on the contribution of diet and bacterial composition in the modulation of TTX concentration are of great importance. Since TTX levels have been known to vary largely between individuals in pufferfishes [43], which was also the case in this study, the unique dietary background and/or bacterial composition of each individual may be a major factor determining TTX accumulation in tissues, thus introducing high levels of individual variation, which remains largely unexplained after accounting for factors such as sex, area, maturity stage, etc. The high unexplained variability in our TTX model (54%) can therefore be attributed to the inevitably high individual variability caused by extrinsic (dietary background) or intrinsic (bacterial composition) factors. These factors are difficult to take into consideration, representing an impediment to developing an effective tool (e.g., a GLM) for predicting precisely the toxin levels in *L. sceleratus*.

### 3.3. TTX Analogues

This is, to our knowledge, the first attempt to analyze variation in TTX analogues’ levels in pufferfishes or other animals. The concentrations of the different TTX analogues were shown to be highly and positively correlated with TTX concentration. In the case of equilibrium analogues (4-epiTTX, 4,9-anhydroTTX), this was to be expected since ΤΤΧ conversion to these analogues has been shown to take place inside the pufferfish body [70]. Moreover, the high correlation between TTX and equilibrium analogues is in accordance with the findings of Nakamura and Yasumoto [71], who showed that the relative concentrations of TTX, 4-epiTTX and 4,9-anhydroTTX are essentially constant in pufferfishes.

On the other hand, the conversion of non-equilibrium analogues (deoxy analogues, 11-norTTX-6-ol) to TTX has never been observed in pufferfishes [16,70]. However, in the present study, concentrations of these analogues were also highly and positively correlated with TTX. This implies that although TTX and non-equilibrium analogues are generally believed to have an exogenous origin [16], bio-transformations between TTX and TTX analogues may also occur inside the body of *L. sceleratus*, probably by endosymbiotic TTX-producing bacteria. Otherwise, the correlations between TTX and these analogues would be weak, as their concentrations would be mainly determined by the (variable) toxins’ composition of ingested preys.

The high correlation of analogue concentrations with TTX implied that the use of relative concentrations (TTX analogue/TTX) was more appropriate to study variations in these compounds in relation to other factors. The GLM analysis showed that the mean relative concentration of 4,9-anhydroTTX was significantly higher in the liver. This observation is in accordance with the findings of Jang and Yotsu-Yamashita [72] who reported that 4,9-anhydroTTX was one of the major TTX analogues in the *Fugu pardalis* liver. Moreover, the highest, albeit not statistically significant, relative concentration of 4-epiTTX was also found in the same tissue. It seems therefore that TTX conversion to the less toxic equilibrium analogues, 4,9-anhydroTTX and 4-epiTTX, might be enhanced in the liver. High relative concentrations of the majority of TTX analogues were also found in skin and gonads, whereas muscles exhibited the lowest relative concentrations of all analogues. In conclusion, muscle is the tissue with lower concentration of both TTX and TTX analogues, which agrees with the conclusion of Noguchi et al. [9] that the muscle of pufferfishes has generally low efficacy to store toxins.

With regard to sex, the relative concentrations of all TTX analogues were significantly higher in females compared to males. This might indicate that TTX biosynthesis and metabolism is enhanced in female fish. Concentrations of certain analogues were also affected by maturity stage. More specifically, the onset of sexual maturation appears to be associated with an increase in 4-epiTTX/TTX, which was significantly lower in virgin fish (maturity stage 0). A differentiation related to maturity stage was also evident for 11-norTTX-6-ol, 11-deoxyTTX, and 5,11/6,11-dideoxyTTX, with higher estimated mean relative concentrations in spawning and post-spawning fish (maturity stage 3 and 4). These findings indicate that the state of maturation may alter the metabolic and/or biosynthetic rate of TTX in *L. sceleratus*. Ikeda et al. [44] have suggested that maturity affects the transfer and/or accumulation of TTX, which might also apply to certain TTX analogues.

### 3.4. Tetrodotoxication Risk

*L. sceleratus* is not included in the list of Japanese edible pufferfishes [3] as TTX levels of its muscle usually exceeds the threshold of 2.2 μg TTX eq g^−1^. The latter concentration has been set as a safety consumption limit for pufferfish flesh in Japan and has also been used to assess the toxicity of *L. sceleratus* samples in the Mediterranean Sea [34,41]. Based on this threshold, the present study highlights the great risk of food poisoning from potential consumption of *L. sceleratus* flesh and reveals the significant effect of fish geographical origin on the severity of that risk. More specifically, the probability of tetrodotoxication was almost two and a half times higher if the fish to be consumed originated from the Libyan Sea compared to Cretan Sea. This finding is of great importance for public safety, as in Southern Crete, there are events of *L. sceleratus* consumption by locals (personal observations). Sabrah et al. [56] and Katikou et al. [34] reported that juvenile fish are generally nontoxic. On the other hand, Leonardo et al. [41] recently reported that juvenile fish can be toxic, as the TTX levels in some tissues were close or above 2 µg g^−1^. In juvenile fish (maturity stage 0) examined in the present study, toxin concentration ranged from 0.05 to 5.02 μg TTX eq g^−1^ in the Cretan Sea and from 1.22 to 11.00 μg TTX eq g^−1^ in the Libyan Sea, meaning that although juveniles can have, on average, lower toxicity levels than adults, they still cannot be considered safe for consumption (based on the specific toxicity threshold of 2.2 μg TTX eq g^−1^). In the Greek seas, juvenile pufferfish are reported to be accidentally caught along with small commercial pelagic fish [41]. With toxicity risk being high regardless of the ontogenetic stage of fish (juvenile or adult), incidental bycatch of pufferfish may pose additional risk for consumers. If not identified correctly and discarded by fishermen, this potential toxic bycatch can end up in the market. In conclusion, flesh consumption of *L. sceleratus* should be considered unsafe regardless of the size, maturity stage, and origin of the fish. Moreover, fishermen should be very careful when sorting their catch and consumers should be properly informed in order to be able to recognize and avoid this toxic species.

Finally, it should be noted that the acceptable levels of TTX in food is a debatable topic. Based on a literature review, the EFSA Panel on Contaminants in the Food Chain (CONTAM) recently proposed the concentration of 44 µg TTX kg^−1^ shellfish meat as a safe limit for consumption [1]. This threshold is much more conservative than the 2.2 μg TTX eq g^−1^, used for the acceptability of pufferfish as food in Japan. If we had used the former toxicity threshold, all muscle samples in our study, except one, would have been characterized as unsafe for consumption.

## 4. Conclusions

This study showed that the type of tissue, geographic area, sex, and maturity stage are significant sources of variation in TTX and TTX analogues’ concentrations. However, the variability explained by the general linear models and the binomial model for tetrodotoxication risk was relatively low due to the large inter-individual variability. The latter could be attributed to the unique dietary background and/or endosymbiotic bacterial composition of each fish. Toxins’ concentration and tetrodotoxication risk is therefore difficult to predict precisely, and silver-cheeked toadfish consumption should be avoided. On the other hand, predicted TTX concentrations from models, such as the GLM developed in this study, could be used to obtain some initial information regarding the probability of harvesting TTX-rich fish. This would be particularly useful in case of an increased interest for commercial exploitation of *L. sceleratus* in the future, as a source of TTX for medical and/or pharmaceutical use [73]. This would require a sufficient amount of TTX in the exploited pufferfish biomass to encourage the development of biorefineries for TTX extraction and the establishment of economically sustainable fisheries in the areas suffering from this invasive species. In turn, increased fishing pressure on *L. sceleratus* would help towards the reduction of its populations and mitigation of its negative environmental impact in the Eastern Mediterranean.

## 5. Materials and Methods

### 5.1. Fish Collection and Measurements

**Figure 5:**
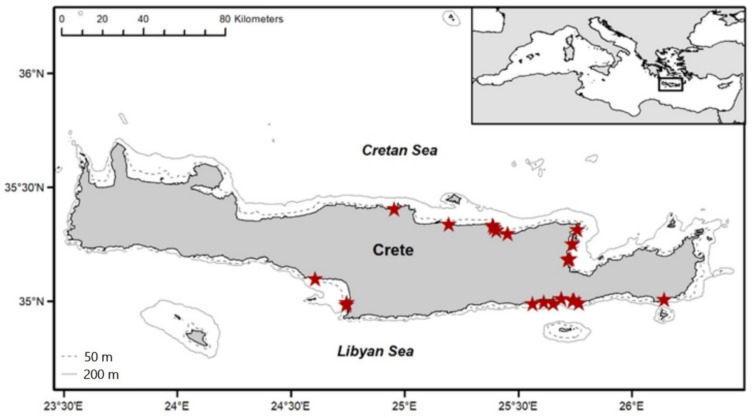
*L. sceleratus* sampling locations.

The *L. sceleratus* samples (see Table 6) were collected off the coasts of the island of Crete, Greece (Figure 5) during the period September 2017–February 2020. In total, 83 specimens, 45 from the Cretan Sea (north) and 38 from the Libyan Sea (south), were fished at depths ranging between 2 and 48 m. Eleven (11) fish were caught using recreational gears (handlines, fishing rods, spearguns) while all other individuals (72) were obtained from the catches of local professional fishers, using nets, long lines, boat seines, and purse seines. The Cretan and Libyan Seas have different ecological and habitat characteristics. More specifically, in the Libyan Sea, waters are warmer, less productive, and the continental shelf is very steep compared to the Cretan Sea [67,68,74]. The number of lessepsian migrants and their abundances, including the abundance of *L. sceleratus*, are higher in the Libyan Sea, where the silver-cheeked toadfish is consistently caught in higher numbers by the local fisheries [75,76,77].

The collected fish were kept in ice and transferred to the laboratory where they were dissected, after measuring their total length (TL, mm) and weight (TW, g). The sex of each specimen was recorded, and the maturity stage of gonads was identified macroscopically using a simplified maturity scale (0: virgin, 1: resting/early developing, 2: maturing, 3: spawning, 4: spent), adapted from the MEDITS maturity stages for Mediterranean bony fish [78] (Supplementary Materials Table S1 and Figure S1). The gastrointestinal tracks of the examined fish were weighted and dissected, while their contents were analyzed to determine diet compositions (further information on the diet analysis are provided in the Supplementary Materials).

### 5.2. Analysis of TTX and Its Analogues

#### 5.2.1. Chemicals and Reagents

A certified reference solution of tetrodotoxin (TTX; ≥98% purity), containing traces of five analogues (11-deoxyTTX, 4,9-anhydroΤΤΧ, 4-epiΤΤΧ, 11-norTTX-6-ol, and 5,6,11-trideoxyTTX) and neat N-methyl-D-glucamine (Internal Standard; ≥99% purity) were purchased from Laboratorio CIFGA S.A. (Lugo, Spain) and Sigma-Aldrich GmbH (Taufkirchen, Germany), respectively. The stock solution of TTX (25.1 ng μL^−1^) and its analogues (0.02 to 2.99 ng μL^−1^), the internal standard working solution (100 ng μL^−1^), and seven calibration standard solutions (TTX: 0.002 to 1.3 ng μL^−1^; TTX analogues: 0.005 pg μL^−1^ to 0.15 ng μL^−1^) were prepared in methanol:water 1:1 solution containing 0.25% acetic acid, and stored at −20 °C until use. N-methyl-D-glucamine was also added in the calibration standard solutions to obtain a final concentration of 0.46 ng μL^−1^. Hydrochloric acid (≥37% purity) was purchased from Honeywell (Seelze, Germany), while all other solvents, including HPLC-grade methanol, acetonitrile, and water (Chromasolv for HPLC; ≥99.9%), as well as formic acid (LiChropur for LC-MS; 98–100% purity), acetic acid (≥99.7% purity), and ammonium formate (HPLC-grade, ≥99% purity) were purchased from Sigma-Aldrich, Taufkirchen, Germany.

#### 5.2.2. Tissue Sample Collection

From each specimen, tissue samples from the gonads (ovaries or testes), liver, muscle, and skin were removed in order to perform the TTX analysis. A total of 332 samples (83 specimen × 4 tissue samples) were collected and analyzed. We assumed that the toxins are homogeneously distributed within each tissue, and, for consistency reasons, we sampled the same part of the organ in each specimen. Muscle and skin samples were dissected from a body area located laterally and behind the dorsal fin, whereas the gonad and liver samples were obtained from the middle part of each organ. In small-bodied individuals, the entire organs (gonads and livers) were used for toxin measurements. Before obtaining a sample, all dissection tools were thoroughly rinsed with water and wiped dry to prevent cross-contamination between the different tissues.

#### 5.2.3. Extraction of TTX and Its Analogues

A piece (1 g) of tissue (muscle, skin, liver, and gonads) was chopped and 0.2 g was collected for the analysis of TTX and its analogues. This small piece was minced and transferred in 2 mL microcentrifuge tube containing 1.5 mL 0.1% acetic acid in water and two stainless-steel beads (5 mm, Qiagen, Hilden, Germany). Tissues were disrupted using a TissueLyser II (Retsch, Qiagen, Hilden, Germany) at 30 Hz for 10 min and centrifuged at 10,000× *g* for 10 min. This procedure was repeated twice and both supernatants (maximum volume 3 mL) were collected in 5 mL polypropylene tubes.

Custom-made Solid-Phase Extraction (SPE) cartridges were prepared by dry-packing 10 mg of polymer-based sorbent (Strata-X-C 33μm polymeric strong cation, Phenomenex, Aschaffenburg, Germany) into 1 mL polypropylene pipette tips, the lower end of which were stoppered with a small piece of wool. Packed cartridges were mounted on a vacuum manifold (VM12 12-port vacuum SPE manifold, Phenomenex, Aschaffenburg, Germany) and conditioned with 500 μL of 0.1% hydrochloric acid in methanol and 500 μL of 0.1% acetic acid in water. Subsequently, a 500 μL aliquot of each sample was loaded onto a SPE cartridge. After a two-step washing procedure with 500 μL of 0.1% acetic acid in water and 400 μL of 0.1% acetic acid in methanol, TTX and its analogues were selectively eluted (>98% of the amount of each compound) using 800 μL of 0.1% hydrochloric acid in water and collected in polypropylene vials. The flow rate during SPE procedure was adjusted to 0.5 drop/s. The eluates were spiked with 3.1 μL of internal standard solution (310 ng in total) and stored at −20 °C until LC-MS/MS analysis.

#### 5.2.4. LC-MS/MS Analysis

All analyses of TTX and its analogues were carried out using an Agilent 1260 Infinity HPLC with binary pump coupled to an Agilent 6460C triple quadrupole mass spectrometer equipped with an Agilent Jet Stream Electrospray source (Agilent Technologies, Waldbronn, Germany). A sample volume of 10 µL was injected into the system, and the chromatographic separation of analytes was achieved on a HILIC column (XBridge BEH Amide XP, 2.1 mm × 100 mm, 2.5 μm particles; Waters corporation, Eschborn, Germany) fitted with a VanGuard pre-column (XBridge BEH Amide XP, 2.1 mm × 5 mm, Waters corporation, Eschborn, Germany) by applying the following binary gradient of solvent A (100% water) and solvent B (95% acetonitrile/water), both containing 3.6 mM formic acid and 2 mM ammonium formate: 10% A for 1 min, from 10% to 50% A in 4 min, hold at 50% A for 1 min, and then back to 10% A for the remaining 7 min (total chromatographic time: 13 min). The column temperature was set at 30 °C and the flow rate was 0.45 mL min^−1^.

The operating parameters of the electrospray ionization source were optimized for TTX analysis, and the optimal conditions were as follows: drying gas temperature 150 °C, drying gas flow rate 10 L min^−1^, sheath gas temperature 380 °C, sheath gas flow rate 12 L min^−1^, nebulizer pressure 40 psi, capillary voltage 2000 V, and nozzle voltage 0 V. The triple quadrupole was operated in the positive ion scan mode using dynamic multiple reaction monitoring (d-MRM) for enhanced selectivity and specificity, and the retention time window (Delta RT) for the detection of analytes was set at 2 min. MRM transitions (one quantitative and one or two confirmatory) were acquired for each compound, the d-MRM parameters of which were optimized and are presented in Table 7. A chromatographic peak was assigned to 6,11- and 5,11-dideoxyTTX based on the relative retention time and the MRM transitions presented by Rambla-Alegre et al. [40]. The concentration of these analogues was semi-quantitatively determined assuming the same analytical response factor as for 11-deoxyTTX. Processing of LC-MS/MS data and quantitation of TTX and its analogues was performed with MassHunter Quantitative Analysis software version B.07.01 (Agilent technologies, Waldbronn, Germany).

#### 5.2.5. Quality Control and Assurance

Internal standard calibration curves were prepared for TTX and its analogues by analyzing a series of seven standard solutions. The calibration curve of TTX was linear at the concentration range 0.002–1.3 ng μL^−1^ and the same was evident for all TTX analogues at concentrations above their detection limit (i.e., 0.03 pg μL^−1^ to 0.15 ng μL^−1^). For all analytes, the regression coefficient R^2^ of the calibration curve was higher than 0.99.

In order to determine detection and quantification limits (DL and QL), a standard solution of TTX and its analogues was prepared at a concentration near the expected detection limit and analyzed seven times. The DL and QL were then calculated as 3.3 and 10 times the standard deviation of the replicate measurements of each analyte, respectively, divided by the slope of the respective calibration curve. The DLs of TTX, 11-deoxyTTX, 4,9-anhydroΤΤΧ, 4-epiΤΤΧ, 11-norTTX-6-ol, and 5,6,11-trideoxyTTX were 0.17, 0.06, 0.81, 0.23, 0.03, and 0.04 pg μL^−1^, respectively (translated to method detection limits of approximately 0.004, 0.001, 0.019, 0.005, 0.001, and 0.001 μg g^−1^), while the respective QLs were 0.51, 0.19, 2.44, 0.69, 0.10, and 0.12 pg μL^−1^, respectively. TTX was detectable in all samples analyzed, while only 6% of the total measurements performed for TTX analogues were below the DL.

To evaluate the efficiency of the extraction procedure, one tissue sample of *L. sceleratus* was subjected to three consecutive extraction cycles, and the extracts were separately analyzed. These results showed that the percentage of TTX in the first extraction was 94%, while only 6% of TTX was extracted during the second cycle. Identical results were obtained for the TTX analogues. A procedure of two extraction cycles was finally adopted in the present study to ensure an extraction efficiency of 99%.

Before the analysis of samples, the SPE protocol adopted for the cleanup of TTX extracts was specifically optimized to attain quantitative recovery of TTX and its analogues. The efficiency of the procedure was evaluated several times using both *L. sceleratus* extracts and standard solutions of TTX. In all cases, the percentage of TTX and its analogues in the collected SPE fraction was higher than 98% and 95%, respectively, implying quantitative recovery of the target analytes. As a result, the measurements made in this study were not subjected to recovery correction. In addition, blank subtraction was not necessary as the target analytes were not detectable in the blank samples analyzed.

#### 5.2.6. Statistical Analysis

For the statistical analyses, non-detected values were substituted by half the detection limit [79]. The strength of the association between TTX and TTX analogues was assessed with correlation analysis. Pearson correlation coefficients were estimated between the concentration of TTX and the concentration of each TTX analogue [80]. To investigate the factors affecting the concentration of TTX, as well as the relative concentration of TTX analogues (i.e., TTX analogue/TTX, see below), we utilized general linear models (GLMs) [81]. The variables tested included the TISSUE (gonad, liver, muscle, and skin), AREA (Cretan Sea and Libyan Sea), SEX (female and male), MATURITY (0, 1, 2, 3, and 4), SEASON (winter, spring, summer, and autumn), and SIZE (total length in mm) of the analyzed specimens. More specifically, the following GLM model was applied for TTX concentration:

log_e_ (TTX) = a + b1 × (TISSUE) + b2 × (AREA) + b3 × (SEX) + b4 × (MATURITY) + b5 × (SEASON) + b6 × log_e_ (SIZE) (1)

where a, b1, b2, …, are the model coefficients. Similar models were also tested for the TTX analogues. The rationale of analyzing the relative concentrations of TTX analogues (TTX analogue/TTX), rather than their absolute concentrations, was that the latter were significantly and highly correlated with the TTX concentration (see Results Section). For all variables having a significant effect in each GLM, least-square means (mean response adjusted for all other variables in the model) were plotted and compared with a posteriori Bonferroni tests.

To assess the overall toxicity of the collected fish and food poisoning risk in case of flesh consumption, the concentration of all toxins (TTX + TTX analogues) in muscle samples were summed after normalizing the levels of TTX analogues against their relative potencies [1]. A sample was then characterized as either toxic and non-toxic, based on a threshold of 2.2 μg TTX eq g^−1^ (equivalent to 10 MU TTX eq g^−1^) [82], above which pufferfish flesh is considered toxic and non-edible in Japan [34]. The toxic samples were further classified as slightly toxic (2.2–22 µg g^−1^), moderately toxic (22–220 µg g^−1^), or extremely toxic (>220 µg g^−1^) based on Yu [83]. To investigate the factors affecting the safety of flesh consumption, a generalized linear model was applied [84]. For the purposes of this analysis, a Bernoulli 0/1-type variable was generated for each sample, by assigning the value 0 to samples having toxin concentration lower than 2.2 µg g^−1^ (assumed safe) and the value 1 to the remainder samples. The obtained Bernoulli variable was assumed to follow a binomial distribution, where the estimated probability is a linear function of the predictor variables. The logit function was used as a link between the linear factor component and the binomial error. The model predictors included the categorical variables AREA, SEX, MATURITY, and SEASON as well as the continuous variable SIZE (entered as main effects). In this way, the probability of a sample being toxic in relation to the above variables was modeled. Model fitting was accomplished by means of “stats” package under the R language environment [85]. Statistical inference was based on the 95% significance level, while probabilities for the levels of each variable, found to be significant, were calculated by averaging over the levels of the remaining predictors.

## Figures and Tables

**Table 1:** Mean (±standard deviation) concentration of TTX and its analogues (μg g^−1^) in *L. sceleratus* tissues, collected from 83 specimens (concentration ranges are shown in parentheses).

Toxins	% Detection Rate in Samples	Tissue			
		Gonads	Liver	Muscle	Skin
TTX	100%	39.85 ± 45.45 (0.30–189.03)	12.18 ± 19.79 (0.04–104.41)	3.76 ± 4.59 (0.02–20.72)	3.87 ± 3.75 (0.12–18.59)
4-epiTTX	100%	3.05 ± 4.71 (0.01–21.59)	1.68 ± 3.29 (ND–15.75)	0.15 ± 0.26 (0.01–1.26)	0.36 ± 0.61 (0.01–4.68)
11-norTTX-6-ol	100%	5.26 ± 8.58 (0.01–47.79)	2.21 ± 5.88 (0.003–39.72)	0.34 ± 0.80 (0.001–5.35)	0.27 ± 0.46 (0.001–2.32)
4.9-anhydroTTX	90%	6.15 ± 9.66 (ND–43.10)	3.09 ± 6.08 (ND–34.63)	0.15 ± 0.20 (ND–1.13)	0.31 ± 0.39 (ND–1.98)
11-deoxyTTX	100%	19.23 ± 40.62 (0.02–281.86)	8.87 ± 26.32 (0.01–176.55)	1.38 ± 3.40 (0.004–21.98)	1.49 ± 2.83 (0.02–18.84)
5,6,11-trideoxyTTX	79%	1.46 ± 5.85 (ND–32.67)	0.02 ± 0.05 (ND–0.33)	0.005 ± 0.04 (ND–0.02)	0.02 ± 0.06 (ND–0.47)
5,11/6,11-dideoxyTTX	96%	2.79 ± 8.01 (ND–46.61)	1.52 ± 6.21 (ND–40.00)	0.11 ± 0.47 (ND–3.82)	0.29 ± 0.70 (ND–4.52)
Total Toxins		77.80 ± 98.73 (0.43–535.78)	29.56 ± 59.92 (0.09–312.95)	5.89 ± 8.49 (0.05–41.47)	6.59 ± 7.35 (0.17–35.05)

ND: not detected.

**Table 2:** Results of the GLM with TTX concentration (log_e_-transformed) of *L. sceleratus* as the dependent variable and TISSUE, AREA, SEX, MATURITY, log_e_(SIZE), and SEASON as explanatory variables.

Effect	F	η^2^	adj. r^2^
TISSUE	49.11 ***	0.24	0.46
AREA	61.87 ***	0.14	
SEX	23.83 ***	0.04	
MATURITY	3.66 **	0.03	
log_e_(SIZE)	3.84 ^ns^		
SEASON	1.66 ^ns^		

η^2^: Eta squared is the ratio of the sum of squares for each effect to the total sum of squares. * *p* < 0.05, ** *p* < 0.01, *** *p* < 0.001, ^ns^ non-significant.

**Table 3:** Results of the GLMs using the relative concentration (log_e_-transformed) of different TTX analogues of *L. sceleratus* as dependent variable and TISSUE, AREA, SEX, and MATURITY as explanatory variables.

Effect	4-epiTTX/TTX		11-norTTX-6-ol/TTX		4,9-anhydroTTX/TTX		11-deoxyTTX/TTX		5,11/6,11-dideoxyTTX/TTX	
	F	η^2^	F	η^2^	F	η^2^	F	η^2^	F	η^2^
TISSUE	27.02 ***	0.17	2.41 ^ns^		47.59 ***	0.29	3.53 *	0.03	15.78 ***	0.11
AREA	0.05 ^ns^		11.46 ***	0.06	4.36 *	0.01	2.86 ^ns^		1.66 ^ns^	
SEX	9.15 **	0.04	18.41 ***	0.07	12.62 **	0.03	4.91 *	0.03	5.14 *	0.03
MATURITY	14.21 ***	0.12	8.35 ***	0.08	2.25 ^ns^		10.71 ***	0.11	9.93 ***	0.09
adj. r^2^	0.31		0.21		0.34		0.17		0.23	

η^2^: Eta squared is the ratio of the sum of squares for each effect to the total sum of squares. * *p* < 0.05, ** *p* < 0.01, *** *p* < 0.001, ^ns^ non-significant.

**Table 4:** Analysis of deviance for the binomial model fit to the Bernoulli-type 0/1 variable of the muscle samples of *L. sceleratus*.

Effect	Residuals d.f.	Residual Deviance	*p*
AREA	81	97.40	<0.001
SEASON	78	95.22	0.510
MATURITY	74	90.12	0.253
SEX	73	89.90	0.697
SIZE	72	88.32	0.210
% of total deviance explained	23.17		

**Table 5:** Mean TTX levels (µg g^−1^) and/or TTX ranges in *L. sceleratus* tissues from various locations in the Mediterranean Sea.

Region	Number of Specimens	Gonads	Liver	Intestine	Skin	Muscle	Analysis Method *	Reference
Aegean Sea	43	0.47–46.30	<0.32–44.15	<0.32–37.60	<0.32–1.40	<0.32–3.47	LC-ESI-CID-MS/MS	[35]
Aegean Sea	1–3		0.42–1.73	6.17	0.39–0.41	0.36–0.43	LC-MS using LTQ-Orbitrap XL	[36]
Northwestern Mediterranean	16	0.43–52.07	ND–46.18	0.07–7.15	0.13–3.43	ND–2.83	LC-MS	[37]
Northeastern Mediterranean	80	0.69–35.60	0.89–21.10	0.79–12.5	2.20–11.80	0.70–5.12	Q-TOF LC-MS	[38]
Eastern Mediterranean	20	0.17–80.00	0.12–25.4	0.13–48.8	0.10–3.30	0.07–3.42	LC-MS	[39]
Western Mediterranean	1	25.95	3.08		1.65	1.01	LC-MS	[40]
Western Mediterranean	1	25.22	5.36		2.08	0.98	LC-HRMS	[40]
Western Mediterranean	1	33.55	28.30		3.50	2.53	mELISA	[40]
North Aegean Sea	2				2.59–2.78	1.39–2.88	Electrochemical MB-based immunosensing tool	[41]
North Aegean Sea	2				1.19–1.24	0.48–2.08	LC-HRMS	[41]
North Aegean Sea	2				2.77–3.18	1.52–2.33	mELISA	[41]
Northern Cyprus Sea	16	0.32–12.87	0.11–13.48	0.29–11.74	0.16–6.54	0.21–8.32	dcELISA	[42]
Cretan and Libyan Sea	83	39.85 ± 45.45 (0.30–189.03)	12.18 ± 19.79 (0.04–104.41)		3.87 ± 3.75 (0.12–18.59)	3.76 ± 4.59 (0.02–20.72)	LC-MS	This study

ND: not detected. * LC-ESI-CID-MS/MS = liquid chromatography electrospray ionization tandem mass spectrometry [35]. Q-TOF LC/MS [45]. LC-HRMS = LC coupled to high-resolution mass spectrometry [40]. mELISA= meleimide-based enzyme-linked immunosorbent assay [46,47]. dcELISA = direct competitive enzyme-linked immunosorbent assay [48].

**Table 6:** Numbers of collected specimens and average total length (TL ranges in parentheses) of *L. sceleratus* by sex and season (quarter). Winter: January–March. Spring: April–June. Summer: July–September. Autumn: October–December.

Season	Sex	Number	TL (mm)
Winter	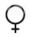	13	296 (174–553)
	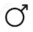	11	247 (149–493)
Spring	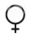	16	532 (226–709)
	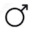	10	556 (443–716)
Summer	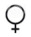	7	513 (450–590)
	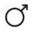	10	522 (312–702)
Autumn	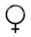	7	429 (270–566)
	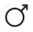	9	412 (247–541)

**Table 7:** Optimized MRM parameters of the quantifier and qualifier transitions that were used for the LC-MS/MS analysis of TTX and its analogues.

Compound	Retention Time (min)	Precursor Ion (*m*/*z*)	Fragmentor	Quantifier			Qualifier		
				Product Ion (*m*/*z*)	Collision Energy	Cell Accelerator Voltage	Product Ion (*m*/*z*)	Collision Energy	Cell Accelerator Voltage
TTX	5.585	320	100	302	24	2	162	36	2
4-epiTTX	5.384	320	100	302	24	2	162	36	2
11-norTTX-6-ol -R, -S	5.187	290	100	272	28	2	162	32	2
4,9-anhydroTTX	4.934	302	100	162	38	2	284	22	2
							256	26	2
11-deoxyTTX	4.908	304	100	286	26	2	162	38	2
							176	36	2
5,11/6,11-dideoxyTTX	4.518	288	100	270	32	2	162	36	2
5,6,11-trideoxyTTX	3.413	272	100	254	22	2	162	44	2
Methyl-glucamine (I.S.)	4.889	196	85	178	11	3	74.1	15	6

## Data Availability

The data sets generated during the current study are available from the authors on reasonable request.

## Supplementary Materials

The following are available online at https://www.mdpi.com/article/10.3390/toxins13120896/s1, Table S1: Maturity stages of L. sceleratus, Figure S1: Photographs illustrating the macroscopic maturity stages of L. sceleratus, Table S2: Frequency of occurrence (FO, %) of prey groups in the gastrointestinal tracks of *L. sceleratus* collected from the Cretan and Libyan Seas. (Reference [86] is cited in the Supplementary Materials.)

## References

[1] Knutsen H.K., Alexander J., Barregård L., Bignami M., Brüschweiler B., Ceccatelli S., Cottrill B., Dinovi M., Edler L., Grasl-Kraupp B. (2017). Risks for public health related to the presence of tetrodotoxin (TTX) and TTX analogues in marine bivalves and gastropods. EFSA J..

[2] Dettbarn W.D., Simpson L.L. (1971). Mechanism of Action of Tetrodotoxin (TTX) and Saxitoxin (STX). Neuropoissons.

[3] Noguchi T., Arakawa O. (2008). Tetrodotoxin-Distribution and accumulation in aquatic organisms, and cases of human intoxication. Mar. Drugs.

[4] Ritson-Williams R., Yotsu-Yamashita M., Paul V.J. (2006). Ecological functions of tetrodotoxin in a deadly polyclad flatworm. PNAS.

[5] Saito T., Noguchi T., Harada T., Murata O., Hashimoto K. (1985). Tetrodotoxin as a Biological Defense Agent for Puffers. Nippon Suisan Gakkai Shi.

[6] Sheumack D., Howden M., Spence I., Quinn R. (1978). Maculotoxin: A neurotoxin from the venom glands of the octopus *Hapalochlaena maculosa* identified as tetrodotoxin. Science.

[7] Matsumura K. (1995). Tetrodotoxin as a pheromone. Nature.

[8] Hanifin C.T., Brodie E.D., Brodie E.D. (2003). Tetrodotoxin levels in eggs of rough-skin newt, *Taricha granulosa*, are correlated with female toxicity. J. Chem. Ecol..

[9] Noguchi T., Arakawa O., Takatani T. (2006). TTX accumulation in pufferfish. Comp. Biochem. Physiol. D 1.

[10] Khora S.S., Isa J., Yasumoto T. (1991). Toxicity of Puffers from Okinawa, Japan. Nippon Suisan Gakkai Shi.

[11] Hwang D.F., Noguchi T., Arakawa O., Abe T., Hashimoto K. (1988). Toxicological Studies on Several Species of Puffer in Taiwan. Nippon Suisan Gakkai Shi.

[12] Hwang D.F., Kao C.Y., Yang H.C., Jeng S.S., Noguchi T., Hashimoto K. (1992). Toxicity of Puffer in Taiwan. Nippon Suisan Gakkai Shi.

[13] Chulanetra M., Sookrung N., Srimanote P., Indrawattana N., Thanongsaksrikul J., Sakolvaree Y., Chongsa-Nguan M., Kurazono H., Chaicumpa W. (2011). Toxic Marine Puffer Fish in Thailand Seas and Tetrodotoxin They Contained. Toxins.

[14] Guardone L., Maneschi A., Meucci V., Gasperetti L., Nucera D., Armani A. (2020). A Global Retrospective Study on Human Cases of Tetrodotoxin (TTX) Poisoning after Seafood Consumption. Food Rev. Int..

[15] Yotsu-Yamashita M. (2001). Chemistry of puffer fish toxin. J. Toxicol. Toxin Rev..

[16] Yotsu-Yamashita M., Abe Y., Kudo Y., Ritson-Williams R., Paul V.J., Konoki K., Cho Y., Adachi M., Imazu T., Nishikawa T. (2013). First identification of 5,11-dideoxytetrodotoxin in marine animals, and characterization of major fragment ions of tetrodotoxin and its analogs by high resolution ESI-MS/MS. Mar. Drugs.

[17] Yasumoto T., Yotsu-Yamashita M. (1996). Chemical and etiological studies on tetrodotoxin and its analogs. J. Toxicol. Toxin Rev..

[18] Shida Y., Arakawa O., Onoue Y., Noguchi T. (1998). LC/MS of Marine Toxin-1. Proceedings of the 46th Annual Conference on Mass Spectrometry.

[19] Kawabata T. (1978). Puffer toxin. The Manual for the Methods of Food Sanitation Tests.

[20] Asakawa M., Shida Y., Miyazawa K., Noguchi T., Calderon L.A. (2012). Instrumental Analysis of Tetrodotoxin. Chromatography-The Most Versatile Method of Chemical Analysis.

[21] Streftaris N., Zenetos A. (2006). Alien marine species in the Mediterranean-the 100 ‘worst invasives’ and their impact. Mediterr. Mar. Sci..

[22] Akyol O., Ünal V., Ceyhan T., Bilecenoglou M. (2005). First confirmed record of *Lagocephalus sceleratus* (Gmelin, 1789) in the Mediterranean Sea. J. Fish Biol..

[23] Regulation 853/2004/EC, 25/6/2004 (2004). Regulation (EC) No 853/2004 of the European Parliament and of the Council of 29 April 2004 Laying Down Specific Hygiene Rules for Food of Animal Origin.

[24] Regulation 854/2004/EC, 25/6/2004 (2004). Regulation (EC) No 854/2004 of the European Parliament and of the Council of 29 April 2004 Laying Down Specific Hygiene Rules for the Organization of Official Controls on Products of Animal Origin Intended for Human Consumption.

[25] Farrag M.M.S. (2014). Fisheries and Biological Studies on Lessepsian Pufferfish, *Lagocephalus sceleratus* (Gmelin, 1789) (Family: Tetraodontidae) in the Egyptian Mediterranean Waters. PhD Thesis.

[26] Bilecenoğlu M., Kaya M., Akalin S. (2012). Range expansion of silverstripe blaasop, *Lagocephalus sceleratus* (Gmelin, 1789), to the northern Aegean Sea. Aquat. Invasions.

[27] Bentur Y., Ashkar J., Lurie Y., Levy Y., Azzam Z.S., Litmanovich M., Golik M., Gurevych B., Golani D., Eisenman A. (2008). Lessepsian migration and tetrodotoxin poisoning due to *Lagocephalus sceleratus* in the eastern Mediterranean. Toxicon.

[28] Chamandi S.C., Kallab K., Mattar H., Nader E. (2009). Human poisoning after ingestion of puffer fish caught from Mediterranean Sea. Middle East J. Anesthesiol..

[29] Kheifets J., Rozhavsky B., Girsh Solomonovich Z., Marianna R., Soroksky A. (2012). Severe Tetrodotoxin Poisoning after Consumption of *Lagocephalus sceleratus* (Pufferfish, Fugu) Fished in Mediterranean Sea, Treated with Cholinesterase Inhibitor. Case Rep. Crit. Care.

[30] Souissi J.B., Rifi M., Ghanem R., Ghozzi L., Boughedir W., Azzurro E. (2014). *Lagocephalus sceleratus* (Gmelin, 1789) expands through the African coasts towards the Western Mediterranean Sea: A call for awareness. Manag. Biol. Invasions.

[31] Ünal V., Bodur H.G., Briand F. (2018). Impacts of pufferfish on human activities in Turkey, Eastern Mediterranean: Special emphasize on *L. sceleratus*. Engaging Marine Scientists and Fishers to Share Knowledge and Perceptions–Early Lessons.

[32] Halim Y., Rizkalla S. (2011). Aliens in Egyptian Mediterranean waters. A check-list of Erythrean fish with new records. Mediterr. Mar. Sci..

[33] Nader M., Indray S., Boustany L. (2012). The Puffer Fish *Lagocephalus sceleratus* (Gmelin, 1789) in the Eastern Mediterranean. East Med Technical Documents 2012.

[34] Katikou P., Georgantelis D., Sinouris N., Petsi A., Fotaras T. (2009). First report on toxicity assessment of the Lessepsian migrant pufferfish *Lagocephalus sceleratus* (Gmelin, 1789) from European waters (Aegean Sea, Greece). Toxicon.

[35] Rodríguez P., Alfonso A., Otero P., Katikou P., Georgantelis D., Botana L.M. (2012). Liquid chromatography-mass spectrometry method to detect Tetrodotoxin and Its analogues in the puffer fish *Lagocephalus sceleratus* (Gmelin, 1789) from European waters. Food Chem..

[36] Bane V., Brosnan B., Barnes P., Lehane M., Furey A. (2016). High-resolution mass spectrometry analysis of tetrodotoxin (TTX) and its analogues in puffer fish and shellfish. Food Addit. Contam. Part A Chem. Anal. Control. Expo. Risk Assess..

[37] Kosker A.R., Özogul F., Durmus M., Ucar Y., Ayas D., Regenstein J.M., Özogul Y. (2016). Tetrodotoxin levels in pufferfish (*Lagocephalus sceleratus*) caught in the Northeastern Mediterranean Sea. Food Chem..

[38] Kosker A.R., Özogul F., Ayas D., Durmus M., Ucar Y., Regenstein J.M., Özogul Y. (2019). Tetrodotoxin levels of three pufferfish species (*Lagocephalus* sp.) caught in the North-Eastern Mediterranean sea. Chemosphere.

[39] Acar C., Ishizaki S., Nagashima Y. (2017). Toxicity of the Lessepsian pufferfish *Lagocephalus sceleratus* from eastern Mediterranean coasts of Turkey and species identification by rapid PCR amplification. Eur. Food Res. Technol..

[40] Rambla-Alegre M., Reverté L., del Río V., de la Iglesia P., Palacios O., Flores C., Caixach J., Campbell K., Elliott C.T., Izquierdo-Muñoz A. (2017). Evaluation of tetrodotoxins in puffer fish caught along the Mediterranean coast of Spain. Toxin profile of *Lagocephalus sceleratus*. Environ. Res..

[41] Leonardo S., Kiparissis S., Rambla-Alegre M., Almarza S., Roque A., Andree K.B., Christidis A., Flores C., Caixach J., Campbell K. (2019). Detection of tetrodotoxins in juvenile pufferfish *Lagocephalus sceleratus* (Gmelin, 1789) from the North Aegean Sea (Greece) by an electrochemical magnetic bead-based immunosensing tool. Food Chem..

[42] Akbora H.D., Kunter İ., Erçetïn T., Elagöz A.M., Çïçek B.A. (2020). Determination of tetrodotoxin (TTX) levels in various tissues of the silver cheeked puffer fish (*Lagocephalus sceleratus* (Gmelin, 1789)) in Northern Cyprus Sea (Eastern Mediterranean). Toxicon.

[43] Endo R. (1984). Toxicological studies on puffer fishes: Comparison of toxicities on the various species. J. Toxicol. Sci..

[44] Ikeda K., Emoto Y., Tatsuno R., Wang J.J., Ngy L., Taniyama S., Takatani T., Arakawa O. (2010). Maturation-associated changes in toxicity of the pufferfish *Takifugu poecilonotus*. Toxicon.

[45] Kosker A.R., Özogul F., Durmus M., Ucar Y., Šimat D.A.V., Özogul Y. (2018). First report on TTX levels of the yellow spotted pufferfish (*Torquigener flavimaculosus*) in the Mediterranean Sea. Toxicon.

[46] Reverté L., de la Iglesia P., del Rio V., Campbell K., Elliot C.T., Kawatsu K., Katikou P., Diogène J., Campàs M. (2015). Detection of Tetrodotoxins in Puffer Fish by a Self-Assembled Monolayer-Based Immunoassay and Comparison with Surface Plasmon Resonance, LC-MS/MS, and Mouse Bioassay. Anal. Chem..

[47] Reverté L., Rambla-Alegre M., Leonardo S., Bellés C., Campbell K., Elliot C.T., Gerssen A., Klijnstra M.D., Diogène J., Campàs M. (2018). Development and validation of a maleimide-based enzyme-linked immunosorbent assay for the detection of tetrodotoxin in oysters and mussels. Talanta.

[48] Zhong Q.P., Huang A.C., Wang B., Dong X. (2011). Development of direct competitive ELISA kit for the direction of tetrodotoxin using HRP labeled antigen. Adv. Mat. Res..

[49] Diener M., Christian B., Ahmed M.S., Luckas B. (2007). Determination of tetrodotoxin and its analogs in the puffer fish *Takifugu oblongus* from Bangladesh by hydrophilic interaction chromatography and mass-spectrometric detection. Anal. Bioanal. Chem..

[50] Jang J.H., Lee J.S., Yotsu-Yamashita M. (2010). LC/MS analysis of tetrodotoxin and its deoxy analogs in the marine puffer fish *Fugu niphobles* from the southern coast of Korea, and in the brackish water puffer fishes *Tetraodon nigroviridis* and *Tetraodon biocellatus* from Southeast Asia. Mar. Drugs.

[51] Kudo Y., Yasumoto T., Konoki K., Cho Y., Yotsu-Yamashita M. (2012). Isolation and structural determination of the first 8-epi-type tetrodotoxin analogs from the newt, *Cynops ensicauda popei*, and comparison of tetrodotoxin analogs profiles of this newt and the puffer fish, *Fugu poecilonotus*. Mar. Drugs.

[52] Nunez-Vazquez E.J., Yotsu-Yamashita M., Sierra-Beltran A.P., Yasumoto T., Ochoa J.L. (2000). Toxicities and distribution of tetrodotoxin in the tissues of puffer fish found in the coast of the Baja California Peninsula, Mexico. Toxicon.

[53] Hwang D.F., Noguchi T. (2007). Tetrodotoxin Poisoning. Adv. Food Nutr. Res..

[54] Noguchi T., Onuki K., Arakawa O. (2011). Tetrodotoxin Poisoning Due to Pufferfish and Gastropods, and Their Intoxication Mechanism. ISRN Toxicol..

[55] Itoi S., Yoshikawa S., Asahina K., Suzuki M., Ishizuka K., Takimoto N., Mitsuoka R., Yokoyama N., Detake A., Takayanagi C. (2014). Larval pufferfish protected by maternal tetrodotoxin. Toxicon.

[56] Sabrah M.M., El-Ganainy A.A., Zaky M.A. (2006). Biology and Toxicity of the Pufferfish *Lagocephalus sceleratus* (Gmelin, 1789) from the Gulf of Suez. Egypt. J. Aquat. Res..

[57] Arakawa O., Hwang D.F., Taniyama S., Takatani T., Ishimatsu A., Lie H.J. (2010). Toxins of Pufferfish That Cause Human Intoxications. Coastal Environmental and Ecosystem Issues of the East China Sea.

[58] Peristeraki P., Lazarakis G., Tserpes G. (2010). First results on the maturity of the lessepsian migrant *Lagocephalus sceleratus* (Gmelin 1789) in the eastern Mediterranean Sea. Rapp. Comm. Int. Mer Médit..

[59] Rousou M., Ganias K., Kletou D., Loucaides A., Tsinganis M. (2014). Maturity of the pufferfish *Lagocephalus sceleratus* in the southeastern Mediterranean Sea. Sex. Early Dev. Aquat. Org..

[60] El-Sayed M., Yacout G.A., El-Samra M., Ali A., Kotb S.M. (2003). Toxicity of the Red Sea pufferfish *Pleuranacanthus sceleratus* ‘El-Karad’. Ecotoxicol. Environ. Saf..

[61] Noguchi T., Jeon J.K., Arakawa O., Sugita H., Deguchi Y., Shida Y., Hashimoto K. (1986). Occurrence of Tetrodotoxin and Anhydrotetrodotoxin in *Vibrio* sp. Isolated from the Intestines of a Xanthid Crab, *Atergatis floridus*. J. Biochem..

[62] Noguchi T., Hwang D.F., Arakawa O., Sugita H., Hashimoto K. (1987). *Vibrio alginolyticus*, a tetrodotoxin-producing bacterium, in the intestines of the fish *Fugu vermicularis vermicularis*. Mar. Biol..

[63] Yasumoto T., Yasumura D., Yotsu M., Michishita T., Endo A., Kotaki Y. (1986). Bacterial Production of Tetrodotoxin and Anhydrotetrodotoxin. Agric. Biol. Chem..

[64] Narita H., Matsubara S., Miwa N., Akahane S., Murakami M., Goto T., Nara M., Noguchi T., Saito T., Shida Y. (1987). *Vibrio alginolyticus*, a TTX-producing Bacterium Isolated from the Starfish *Astropecten polyacanthus*. Nippon Suisan Gakkai Shi.

[65] Simidu U., Noguchi T., Hwang D.F., Shida Y., Hashimoto K. (1987). Marine Bacteria Which Produce Tetrodotoxin. Appl. Environ. Microbiol..

[66] Vlamis A., Katikou P., Rodriguez I., Rey V., Alfonso A., Papazachariou A., Zacharaki T., Botana A.M., Botana L.M. (2015). First detection of tetrodotoxin in greek shellfish by UPLC-MS/MS potentially linked to the presence of the dinoflagellate. Prorocentrum Minim. Toxins.

[67] Tsimenides N., Tserpes G., Machias A., Kallianiotis A. (1991). Distribution of fishes on the Cretan shelf. J. Fish Biol..

[68] Bosc E., Bricaud A., Antoine D. (2004). Seasonal and interannual variability in algal biomass and primary production in the Mediterranean Sea, as derived from 4 years of SeaWiFs observations. Glob. Biochem. Cycles.

[69] Auawithoothij W., Noomhorm A. (2012). *Shewanella putrefaciens*, a major microbial species related to tetrodotoxin (TTX)-accumulation of puffer fish *Lagocephalus lunaris*. J. Appl. Microbiol..

[70] Kono M., Matsui T., Fur ukawa K., Takase T., Yamamori K., Kaneda H., Aoki D., Jang J.H., Yotsu-Yamashita K. (2008). Examination of transformation among tetrodotoxin and its analogs in the living culturd juvenile puffer fish, kusafugu, *Fugu nipholbes* by intramuscular administration. Toxicon.

[71] Nakamura M., Yasumoto T. (1985). Tetrodotoxin Derivatives in Puffer Fish. Toxicon.

[72] Jang J., Yotsu-Yamashita M. (2006). Distribution of tetrodotoxin, saxitoxin, and their analogs among tissues of the puffer fish *Fugu pardalis*. Toxicon.

[73] Jal S., Khora S.S. (2015). An overview on the origin and production of tetrodotoxin, a potent neurotoxin. J. Appl. Microbiol..

[74] Stergiou K.I., Christou E.D., Georgopoulos D., Zenetos A., Souvermetzoglou C. (1997). The Hellenic Seas: Physics, Chemistry Biology and Fisheries. Oceanogr. Mar. Biol..

[75] Peristeraki P., Tserpes G., Biyakis S., Kostopoulou V., Anezaki E., Vala E. Observations on the expansion pattern of the invasive *Lagocephalus sceleratus* around Crete: Interactions with coastal fisheries. Proceedings of the MARBIGEN Conference.

[76] Peristeraki P., Skarvelis K., Giannakaki A., Tambakakis K., Tserpes G. Preliminary results on the abundance of alien species in the coastal fisheries of Crete. Proceedings of the 11th Panhellenic Symposium on Oceanography and Fisheries.

[77] Kiparissis S., Peristeraki P., Tambakakis K., Kosoglou I., Doudoumis V., Batargias C. (2018). Range expansion of a restricted lesseptian: Westbound expansion breakthrough of *Lagocephalus spadiceus* (Richardson, 1844) (Actinopterygii: Tetraodontidae). Bioinvasions Rec..

[78] Follesa M.C., Carbonara P. (2019). Atlas of the Maturity Stages of Mediterranean Fishery Resources. Studies and Reviews No. 99.

[79] Hites R.A. (2019). Correcting for censored environmental measurements. Environ. Sci. Technol..

[80] Zarr J.H. (1999). Biostatistical Analysis.

[81] Chambers J.M., Chambers J.M., Hastie T.J. (1992). Linear Models. Chapter 4 of Statistical Models in S.

[82] Horie M., Kobayashi S., Shimizu N., Nakazawa H. (2002). Determination of tetrodotoxin in puffer-fish by liquid chromatography-electrospray ionization mass spectrometry. Analyst.

[83] Yu C.F. (2003). A Comprehensive Study of the Hong Kong Pufferfishes and Their Toxins. Ph.D. Thesis.

[84] McCullagh P., Nelder J.A. (1989). Generalized Linear Models.

[85] R Core Team (2020). R: A Language and Environment for Statistical Computing.

[86] Agresti A. (2007). An Introduction to Categorical Data Analysis.

