# Supplementary material for: Keeping Lagocephalus sceleratus off the Table: Sources of Variation in the Quantity of TTX, TTX Analogues, and Risk of Tetrodotoxication
Source: Toxins (Basel). 2021 Dec 13;13(12):896. doi: 10.3390/toxins13120896 (PMC8706384; doi:10.3390/toxins13120896)
Supplement: Supplementary file 1 [file toxins-13-00896-s001.zip › toxins-1429060-supplementary.pdf]

toxins MDPI

Supplementary Materials: Keeping *Lagocephalus sceleratus* off the Table: Sources of Variation in the Quantity of TTX, TTX Analogues, and Risk of Tetrodotoxication

Georgios Christidis, Manolis Mandalakis, Thekla Anastasiou, George Tserpes, Panagiota Peristeraki and Stylianos Somarakis

A. Maturity stages of *L. sceleratus*

Table S1. Description of *L. sceleratus* macroscopic maturity stages.

Gender	Gonad Aspect	Maturity State	Stage	Figure S1
Female	Small reddish ovaries shorter than 1/3 of the body cavity. Oocytes not visible with the naked eye.	Virgin	0	0a
Male	Thin and whitish testes shorter than 1/3 of the body cavity.			0b
Female	Pinkish ovaries. Size: 1/3 to 1/2 of the body cavity. Oocytes not visible with the naked eye.	Resting/Early developing	1	1a
Male	Whitish/pinkish testes. Size: 1/3 to 1/2 of the body cavity.			1b
Female	Pinkish ovaries with granular appearance. Size: 1/2 to 2/3 of the body cavity. Blood vessels visible. Oocytes visible with the naked eye.	Maturing	2	2a
Male	Whitish/pinkish testes. Size: 1/2 to 2/3 of the body cavity. Blood vessels visible. Under light pressure, sperm is not expelled.			2b
Female	Ovaries orange-pink with conspicuous blood vessels. Size: 2/3 to full length of body cavity. Large oocytes are clearly visible.	Spawning	3	3a
Male	Whitish-creamy testes. Size: 2/3 to full length of the body cavity. Blood vessels visible. Under light pressure sperm might be expelled.			3b
Female	Bloodshot and flaccid ovaries shrunken to about 1/2 the length of the body cavity.	Spent	4	4a
Male	Bloodshot and flabby testes shrunken to about ½ the length of the body cavity.			4b

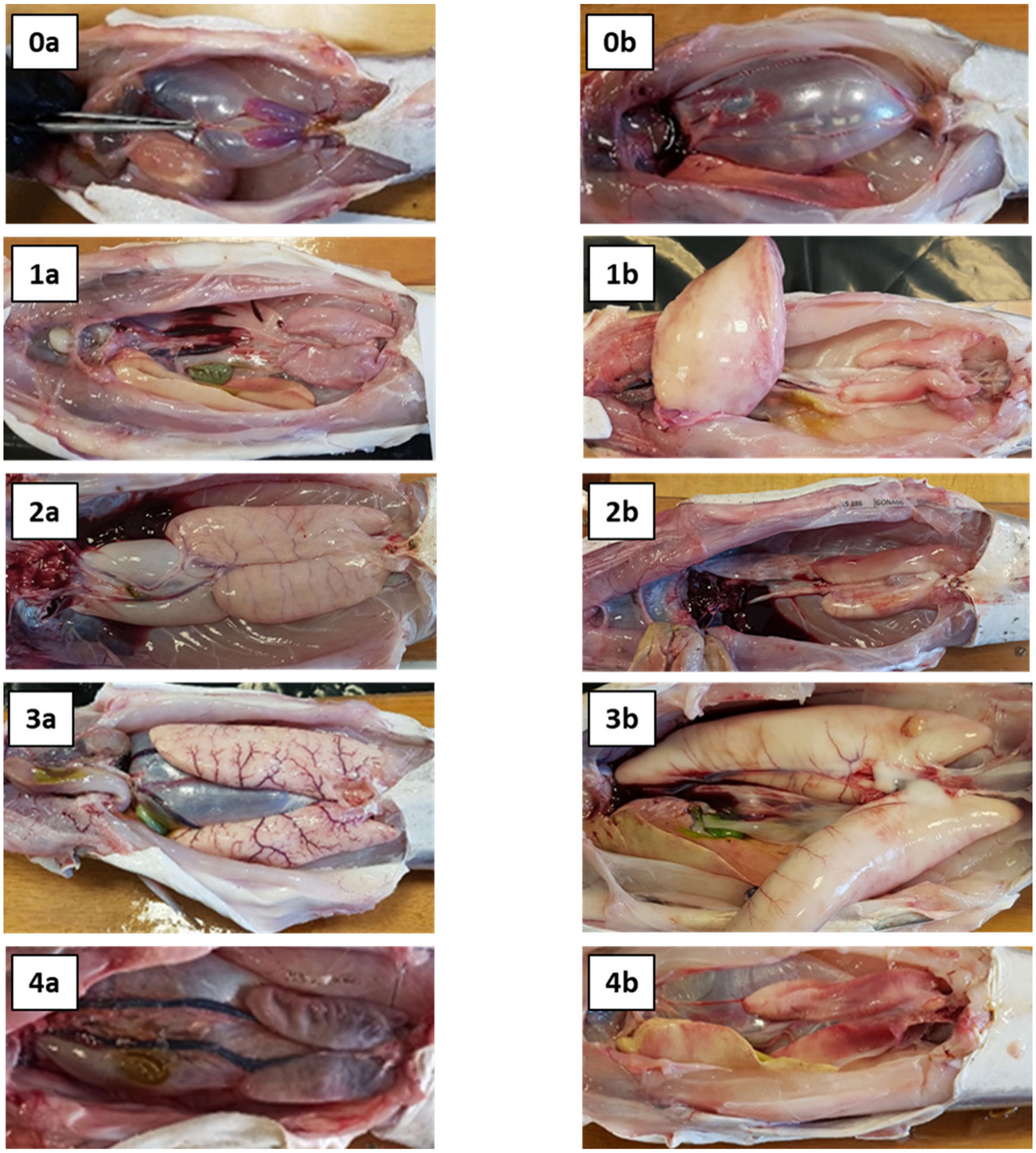

Figure S1. Photographs illustrating the macroscopic maturity stages of female (**a**) and male (**b**) L. sceleratus. 0: Virgin, 1: Resting/Early developing, 2: Maturing, 3: Spawning, 4: Spent.

B. Diet examination of *L. sceleratus*

Gastrointestinal tracks of the examined fish were dissected and their contents were analyzed to determine diet compositions. The presence/absence of gastrointestinal content was recorded and each ingested food item (prey) was identified to broad taxonomic groups (fish, cephalopods, crustaceans, echinoderms, bivalves, gastropods, other invertebrates and photosynthetic organisms). Frequency of occurrence (FO) of each prey-group was estimated as follows:

$$FO\% = \sum \frac{ni}{n} * 100$$

where ni represents the number of fish with i group of prey and n is the total number of fish with food in their gastrointestinal track.

The difference in diet composition between pufferfish originating from the Cretan and Libyan Seas was tested using the Pearson's Chi-square test applied on the prey's frequency of occurrence matrix [86].

Seventy-one (71) out of the 83 gastrointestinal tracks analyzed, contained one or more ingested items, whereas 12 tracks were found empty. Based on the *FO* index, the main preys of L. sceleratus were fish (76%), crustacea (29.6%), photosynthetic organisms (21.1%) and cephalopods (16.9%), while echinoderms (5.6%), bivalves (4.2%), gastropods (4.2%) and other invertebrates (2.8%) occurred less frequently (Table S2).

The difference in diet composition between the specimens collected from the Cretan and the Libyan Sea was statistically significant (df = 8, chi-square = 21.75, $p < 0.01$).

Table S2. Frequency of occurrence (*FO*, %) of prey-groups in the gastrointestinal tracks of *L. sceleratus* collected from the Cretan and Libyan Seas.

Gastrointestinal contents	Cretan Sea	Libyan Sea	Total
**Food items**			
Fish	35.2	40.8	76
Crustacea	18.3	11.3	29.6
Cephalopods	15.5	1.4	16.9
Photosynthetic organisms	11.3	9.8	21.1
Echinoderms	1.4	4.2	5.6
Bivalves	1.4	2.8	4.2
Gastropods	0	4.2	4.2
Other invertebrates	1.4	1.4	2.8
Unidentified	2.8	7.0	9.8

References

86. Agresti, A. *An Introduction to Categorical Data Analysis*, 2nd ed.; John Wiley & Sons, New York, USA, 2007.
